# Dermatitis Herpetiformis in Celiac Disease: A Systematic Review and Meta‐Analysis

**DOI:** 10.1002/ueg2.70259

**Published:** 2026-07-07

**Authors:** Honoria Ocagli, Chiara Monachesi, Giacomo Berti, Ileana Baldi, Fabiana Zingone, Cristina Canova

**Affiliations:** ^1^ Unit of Biostatistics, Epidemiology and Public Health Department of Cardio‐Thoraco‐Vascular Sciences and Public Health University of Padova Padua Italy; ^2^ Unit of Gastroenterology Azienda Ospedale Università Padova Padua Italy; ^3^ Department of Surgery, Oncology and Gastroenterology University of Padua Padua Italy

**Keywords:** celiac disease, dermatitis herpetiformis, iga deposits, meta‐analysis, skin diseases

## Abstract

Dermatitis herpetiformis (DH) shares a pathophysiological basis with celiac disease (CD), but its epidemiology remains poorly defined. This systematic review and meta‐analysis estimated the prevalence of DH among CD patients. Following the PRISMA 2020 guidelines, PubMed, Embase, Web of Science, and Scopus were searched. Observational studies with extractable DH data in CD patients were pooled using a random‐effects model, with subgroup and meta‐regression analyses. The risk of bias was assessed using the JBI Checklist. Of 7271 records, 24 studies published between 1996 and 2023, comprising 73,905 patients with celiac disease and 2203 DH cases (crude prevalence: 3.0%), met the inclusion criteria. Pooled DH prevalence in CD was 6.8% (95% CI: 5.0–9.3). When restricting the analysis to nonselected populations, the pooled prevalence was 6.7% (95% CI: 4.7–9.6). Prevalence was lower in pediatric CD (2.6%, 1.4–4.5) than in adults (8.6%, 5.8–12.6). Gender‐specific prevalence, based on 5 of the 24 studies and not directly comparable with the overall estimate, was 12.9% (7.0–22.7) in males and 9.6% (3.7–22.9) in females. Sensitivity analyses yielded comparable estimates across all models (range: 6%–9%), indicating that the results were consistent across different methodological assumptions and not driven by specific study characteristics. Given the very high heterogeneity (I^2^ = 98.9%), the pooled estimate should be interpreted as an average across diverse settings rather than a single generalizable figure. These findings emphasize the need for standardized diagnostic approaches and more consistent reporting in clinical settings.

**Trial Registration:**

PROSPERO (CRD42023444060)

## Key Summary

- Summarize the established knowledge on this subject:
  ◦ Dermatitis herpetiformis (DH) is a cutaneous manifestation of celiac disease (CD) that shares the same autoimmune basis, yet its epidemiology remains poorly defined. Previous studies have reported inconsistent prevalence estimates due to heterogeneous populations, diagnostic criteria, and study designs.
- What are the significant and/or new findings of this study:
  ◦ This systematic review and meta‐analysis provides the most comprehensive estimate to date, showing that DH affects approximately 7% of CD patients, with a lower proportion in children (2.6%) than in adults (8.6%) and slightly higher rates in males. This study underscores significant heterogeneity across settings and highlights the importance of standardized diagnostic criteria.

## 1 Introduction

Celiac disease (CD) is a chronic autoimmune disorder triggered by the ingestion of gluten in genetically predisposed individuals [1]. It is one of the most frequent lifelong diseases, affecting approximately 1%–2% of the general population worldwide [2].

Dermatitis herpetiformis (DH) is a cutaneous manifestation of CD, characterized by a blistering rash and the presence of pathognomonic IgA deposits in the skin. First described by Duhring in 1884, this condition was initially categorized alongside other disorders such as pemphigus and erythema multiforme. In the 1970s, studies revealed that individuals with DH often exhibited intestinal changes similar to those seen in CD, shared the same genetic predisposition, and a gluten‐free diet was shown to improve skin symptoms, albeit at a slower rate compared to gastrointestinal symptoms [3].

The pathogenesis of DH, which involves a complex inflammatory network along the gut‐skin axis, is still only partially understood. In the past 3 decades, significant research has led to the identification of epidermal transglutaminase as the primary autoantigen in DH and to a detailed understanding of the inflammatory microenvironment that drives the development of skin lesions [3].

Earlier studies on DH indicated a clear male predominance, but recent large studies in adults have found a nearly equal male‐to‐female ratio, contrasting with the female predominance observed in CD [4, 5, 6, 7, 8]. DH can develop at any age, although it mainly affects adults, particularly between the third and fourth decades of life, characterized by a symmetrical rash and severe itching, often localized to the elbows, knees, and buttocks [4, 5, 6, 7, 8, 9]. DH is a complex condition in which skin lesions represent a clue to the identification of gluten‐sensitive enteropathy [5].

Currently, the DH‐to‐CD prevalence is 1:8^4^. In recent decades, the overall incidence of DH has notably declined, while the incidence of CD has been on the rise, probably because of improved awareness of CD among both healthcare providers and patients, along with the widespread use of case‐finding approaches used for CD^4^. This has led to the early identification of patients with asymptomatic or potential CD.

Dermatitis herpetiformis has also been hypothesized to develop as a long‐term complication of untreated or undiagnosed celiac disease, potentially emerging after prolonged gluten exposure and delayed diagnosis of enteropathy [10]. This perspective may partly explain the lower frequency observed in pediatric cohorts and the predominance in adults.

While CD diagnosis follows standardized guidelines, DH is usually diagnosed clinically based on typical lesions in patients with confirmed CD. When DH is suspected first, skin biopsy with direct immunofluorescence, the diagnostic gold standard, is seldom performed, with diagnosis often relying on CD serology and intestinal damage evidence [11].

Evidence on DH prevalence in CD is limited, hindering an accurate assessment of disease burden and risk stratification. This systematic review and meta‐analysis aim to provide a precise estimate of DH prevalence in CD, explore differences by age and sex, and assess DH prevalence in the general population.

## 2 Materials and Methods

The study followed the Preferred Reporting Items for Systematic reviews and Meta‐Analyses (PRISMA) 2020 guidelines [12]. The review was prospectively registered within PROSPERO (CRD42023444060). The registration covers a broader research framework that addresses multiple epidemiological aspects of dermatitis herpetiformis and celiac disease. The completed PRISMA 2020 Checklist is provided in Supporting Information S2. The present manuscript reports a predefined objective related specifically to the prevalence of DH among CD patients.

### 2.1 Eligibility Criteria

The inclusion criteria were the following: observational studies, either cross‐sectional or cohort in design, and randomized controlled trials that reported the number of DH cases within samples of patients diagnosed with CD. DH status was assessed at diagnosis of CD when available; however, in several studies, DH could be identified during follow‐up or retrospectively from clinical records. Although clinical cohorts routinely document this, administrative databases may record DH only when it is the main reason for the visit, potentially missing incident cases. Only studies providing extractable numerical data for both the numerator (number of DH cases) and denominator (total CD population) were included in the analysis. Reviews, editorials, conference abstracts, and case reports were excluded. Language restrictions were not applied. To reduce bias introduced by outdated diagnostic approaches, only studies published from 1990 onward were considered eligible for the prevalence of DH in the general population.

### 2.2 Information Sources

We systematically searched four electronic bibliographic databases: Embase (through Ovid), PubMed (Medline), Web of Science, and Scopus. All searches were initially conducted on July 3, 2023, and were updated on February 9, 2025. No restrictions were applied on language or publication type in the search strategy.

### 2.3 Search Strategy

The search strategy was designed to identify studies reporting on DH, using both controlled vocabulary (e.g., MeSH terms in PubMed and Emtree terms in Embase) and free‐text keywords. The searches were adapted for each database. Synonyms such as “Duhring disease” were included in all databases to maximize sensitivity. See Supporting Information S1 (Table S1) for the full search strategy.

### 2.4 Study Selection and Extraction

Title/abstract and full‐text screening were conducted within the same PROSPERO registration (CRD42023444060), which included multiple predefined review questions. Therefore, a substantial proportion of the full texts retrieved were eligible under the registered protocol but did not address the specific objective of the present manuscript (prevalence of dermatitis herpetiformis among patients with celiac disease) and were reassigned to other predefined questions within the same registration.

The study selection was conducted independently by three reviewers (GB, CM, and CC) using the Covidence platform (Covidence systematic review software, Veritas Health Innovation, Melbourne, Australia. Available at www.covidence.org). Disagreements were resolved by consensus or adjudicated by a fourth reviewer, an expert in CD (FZ). Full texts were retrieved for potentially eligible studies, and inclusion was confirmed according to predefined criteria.

From each included study, we extracted: author, publication year, country or geographic region, number of CD patients, number of DH patients, sex‐stratified results (where available), age group classification (pediatric, adult, mixed), and any additional stratification (subclinical populations). For studies with multiple strata or time points, each eligible data point was considered separately. The study by Grodé et al. [13] was split into two observations reflecting data from 2006 to 2016. For all included studies, the total number of CD patients was used as the denominator and the number of DH cases as the numerator.

### 2.5 Data Synthesis and Statistical Analysis

The primary effect measure was the proportion of patients with DH among all CD patients, expressed as a pooled proportion. Meta‐analyses were conducted using the *metaprop* function of the meta package in R (version 8.0–2), applying a generalized linear mixed model (GLMM) with logit transformation to account for the binomial distribution of the data and to avoid ad hoc continuity corrections. GLMMs have been shown to reduce bias and mean squared error while improving coverage probabilities compared to traditional two‐step methods based on transformed proportions [14]. Analyses were performed under a random‐effects framework. Between‐study variance between studies (tau2) was estimated directly within the GLMM model structure. For all analyses, 95% confidence intervals (CI) were calculated on the logit scale and back‐transformed to the proportion scale for interpretation.

### 2.6 Subgroup and Meta Regression Analyses

Subgroup analyses were conducted by age group (pediatrics, adults), and gender (female, male). Differences between subgroups were formally tested using the Q‐test for subgroup differences, whose test statistic follows a chi‐squared distribution (χ^2^) [15]. Meta regression was performed within the GLMM to explore the influence of moderators such as year of publication, age group (mixed vs. pediatrics), and geographic region (Northern, Western, Eastern Europe, Other) [14]. Variables were included in univariable models, and coefficients were reported with 95% confidence intervals and *p*‐values.

#### 2.6.1 Sensitivity Analysis

Sensitivity analyses were conducted to assess the robustness of the findings of the pooled prevalence estimates and to explore the potential impact of methodological and population‐related sources of bias. The following analyses were predefined: (1) Excluding studies with undefined or unclear outcome definition, including those lacking explicit diagnostic criteria or relying on self‐reported or unspecified methods; (2) Excluding studies conducted in selected populations, such as subclinical celiac disease, symptomatic‐only cohorts, or other restricted subgroups; (3) Excluding registry‐based (administrative) data, given the potential for misclassification and limited diagnostic detail; (4) Excluding the largest study (Lebwohl et al. [16]), to evaluate the influence of a single large dataset on the pooled estimates.

### 2.7 Risk of Bias Assessment

Risk of bias was evaluated using the Joanna Briggs Institute (JBI) *Checklist for Prevalence Studies* [17], a validated tool designed to assess the methodological quality of studies reporting prevalence data. Each item was assessed as “Yes,” “No”, or “Unclear” based on pre‐specified criteria. The assessment was performed independently by two reviewers (CC and FZ), and any discrepancies were resolved through discussion. This structured approach allowed for a consistent evaluation of methodological quality throughout the included literature.

### 2.8 Reporting Bias

Small‐study effects were explored through visual inspection of a funnel plot. In meta‐analyses of proportion studies with extreme outcomes, publication bias plots may produce asymmetry due to statistical artifacts rather than true bias [18].

## 3 Results

**FIGURE 1:**
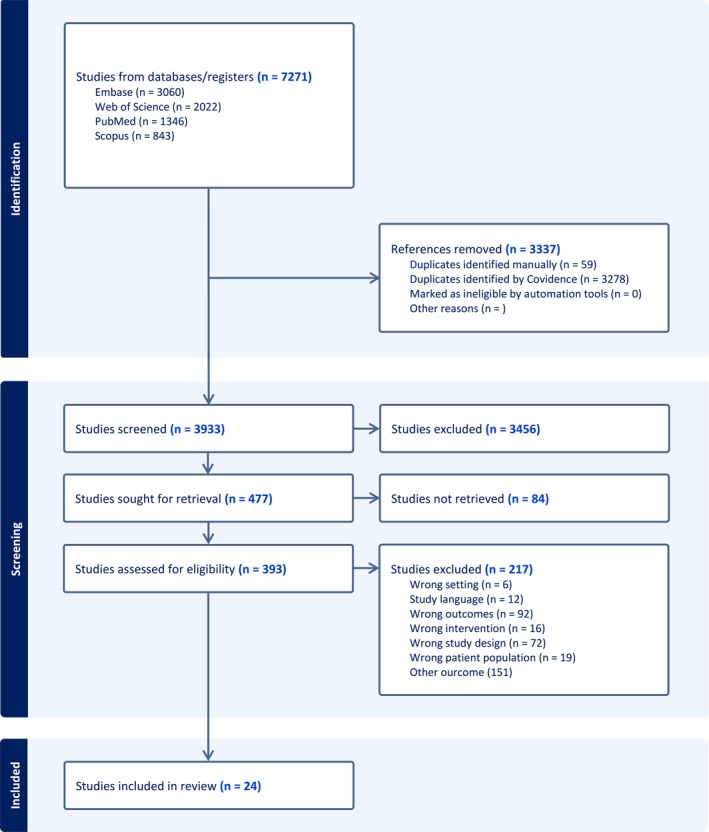
PRISMA flowchart.

A total of 7271 records were identified through database searches (Figure 1). After removing 3337 duplicates, 3933 titles and abstracts were screened. Of these, 3456 were excluded based on their irrelevance to the research question. Full texts were retrieved for 477 records. Of these, 393 full‐text articles were assessed for eligibility for the different questions. Among them, 218 were excluded because they addressed other predefined questions within the same registered project or did not meet the inclusion criteria for any of them. 24 studies met the inclusion criteria and were included in the qualitative and quantitative synthesis for the prevalence analysis, and an additional 4 studies reported incidence estimates of DH in the general population. Grode et al. and Hawkes et al. contributed more than one eligible data stratum and were therefore entered as separate observations in the meta‐analysis.

### 3.1 Studies Characteristics

The characteristics of the included studies are presented in Table 1. The included studies exhibit considerable heterogeneity in terms of design, setting, geography, and population characteristics. Most of the studies were conducted in European countries, including Italy, Finland, Germany, and Denmark, but the sample also includes data from South America (Brazil), North America (USA), Asia (Pakistan, Iran), and Central Europe. Study designs varied and included cross‐sectional studies and retrospective cohorts, with the majority conducted in secondary care settings. A few studies utilized population‐based registries [13], while others relied on hospital databases, outpatient clinics, or community‐based interviews [19].

**TABLE 1:** Characteristics of the included studies.

Author—year	Population	Country	Study design	Care setting	Study period	Age group	CD sample	DH cases	*N* pediatrics	*N* female patients CD	*N* female patients DH	Type of diagnosis CD	Type of diagnosis DH	Age overall
Bari—2023 [19]	CD	Iran	Interview	Community	2018–2021	Mixed	140	15	56	96				
Bottaro—1999 [20]	CD subclinical	Italy	Cross‐sectional study	Secondary	1990–1994	Mixed	798	73		702		Histological findings	Clinical (extraintestinal manifestations)	15.8 ± 13.4
Collin—2007 [10]	CD (people who receive reimbursement for dietary cost)	Finland	Cross‐sectional study	Primary and secondary	2001–2002	> 16 years	1936	333		1355	140	Biopsy or serologic tests	Skin biopsy with direct immunofluorescence (granular iga deposition)	
Delcò—1999 [21]	CD among military	USA	Case‐control	Primary and secondary	1986–1995	Adults	458	34		17		Diagnosis in patient treatment file	Clinical	63.8 ± 12.4
De Freitas—2002 [22]	CD	Brazil	Cross‐sectional study	Secondary	1972–2002	> 15 years	48	2		32		Small‐bowel histological findings		41 (15–68)
Dev—2021 [23]	CD	Pakistan	Cross‐sectional study	Tertiary hospital	2020–2021	Adults	300	48		141	18			39 ± 8
Dibiase—2021 [24]	CD symptomatics	Italy	Cross‐sectional study	Tertiary	2004–2014	Pediatrics	340	18		223		Y CD serology (anti‐ttg/EMA)‐positive patient had to be confirmed histologically by an EGD with duodenal biopsy	Clinical manifestations	
Giorgetti—2008 [25]	CD subclinical	Italy	Cross‐sectional study	Secondary	1988–2007	Adults	195	18				Serology/biopsy	Extraintestinal markers	27.9 (15–65)
Grode—2018 [13]	CD	Denmark	Population‐based study	Registry	1980–2016	Mixed	4171	124		2579		Icd from adminstrative sources	Autoimmune comorbidities	40.3 ± 28.9
Grode—2018 [13]	CD	Denmark	Population‐based study	Registry	1977–2016	Mixed	10,285	348	3395	6839		Icd from adminstrative sources		37.0 ± 26.7
Hawkes—2000 [26]	CD	UK	Retrospective case‐finding study	Secondary	1987–1995	Pediatrics	27	0					Clinical	5.3
Hawkes—2000 [26]	CD	UK	Retrospective case‐finding study	Secondary	1987–1995	Mixed	137	19	27	98	6		Clinical	
Häuser—2006 [27]	CD	Germany	Survey	Community	2005	Adults	446	41		348		Biopsy		49.2 (33–59)
Kotze—2009 [28]	CD	Brazil	Retrospective	Primary	2011–2016	Mixed	157	18	23	125	14	Histological findings and serologic auto‐antibodies markers		
Khan—2013 [29]	CD	Pakistan	Cross‐sectional study	Secondary	2009–2011	Mixed	52	1	33	27				20.19 ± 10.68
Kotze—2018 [30]	CD	Brazil	Cross‐sectional study	Primary	2011–2016	Mixed	213	26	33	161	15			
Lebwohl—2021 [16]	CD	Sweden	Retrospective cohort	Secondary	1969–2016	Mixed	49,744	746					ICD codes	
Lima—2019 [31]	CD	Brazil	Cross‐sectional study	Primary	2000–2017	Adults	240	45		160	33	Clinical complaints, positive autoantibodies (TTG and/or EMA), and confirmation by histological findings		
Papp—2008 [32]	CD	Hungary	Cross‐sectional study	Secondary	2006–2007	Mixed	712	111				Biopsy proven	Direct immunofluorescence of skin biopsy	17* (2–85)
Riestra—2006 [33]	CD	Spain	Cross‐sectional study	Tertiary	1990–2004	Adults	22	5		18		Biopsy		50* (19–77)
Riznik—2019 [34]	CD symptomatics	Central Europe	Cross‐sectional study	Secondary	2017	Pediatrics	393	7	225					
Schiepatti—2022 [35]	CD	Italy	Single‐center retrospective study longitudinal	Secondary and tertiary	2000–2020	Adults	248	16		186		Biopsy and positive endomysial antibodies (ema) and/or tissue transglutaminase antibodies tested while on a gluten containing diet	Clinical	
Sorensen—1996 [36]	CD	Denmark	Cross‐sectional study	Secondary	1977–1992	Adults	896	17		552	10			
Szaflarska‐Poplawska—2009 [37]	Potential CD	Poland	Cross‐sectional study	Secondary	2002–2009	Mixed	30	2	23	18				12.6 (3–28)
Volta—2014 [38]	CD	Italy	Cross‐sectional study	Secondary	1998–2012	Adults	770	31		599			Associated disorder	36* (18–78)
Zingone—2009 [39]	Untreated CD	Italy	Cross‐sectional study	Tertiary	1975–2009	Adults	1147	105		924	64	Duodenal biopsy, auto‐antibodies serum	Medical examination and dermatologist consultation	

The study periods spanned from as early as 1969 [16] to as recent as 2023 [23]. Sample sizes ranged widely, from as few as 22 CD patients [33] to more than 49,000 [16]. The age distribution also varied, with some studies focused exclusively on pediatric populations [24, 26, 34], while others included adults or mixed‐age groups. The gender distribution and diagnostic criteria also differed: several studies applied biopsy‐confirmed diagnoses, while others used serological markers (e.g., EMA, anti‐tTG) or administrative ICD codes.

### 3.2 Overall Prevalence

Overall, the included studies comprised 73,905 patients with celiac disease, of whom 2203 were diagnosed with dermatitis herpetiformis (crude prevalence: 3%). The pooled prevalence estimates from the primary, subgroup, and sensitivity analyses are summarized in Table 2.

**TABLE 2:** Estimates of the meta‐analyses.

Type		*N* studies	Estimate (95% CI)
Overall		24	0.068 (0.050–0.093)
Sensitivity	Excluding undefined/unclear outcome	17	0.063 (0.044–0.090)
	Excluding selected population	18	0.067 (0.047–0.096)
	Excluding registry‐based data	20	0.088 (0.067–0.115)
	Excluding large study^a^	23	0.078 (0.057–0.103)
Subgroup	Pediatrics	5	0.026 (0.014–0.045)
	Adults	12	0.086 (0.058–0.126)
	Male	5	0.129 (0.070–0.227)
	Female	5	0.096 (0.037–0.229)

^a^ Lebwohl et al. [16], *n* = 49,744.

**FIGURE 2:**
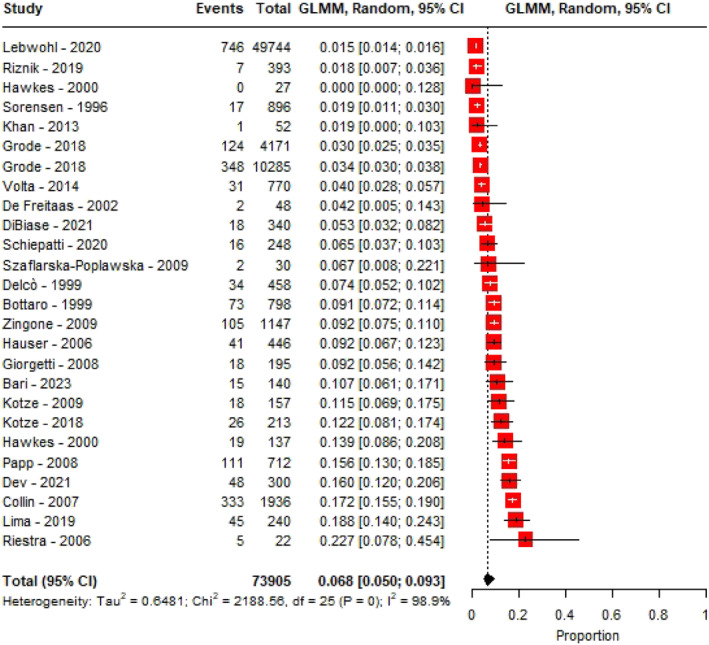
Forest plot shows the overall pooled proportion of dermatitis herpetiformis among patients with celiac disease.

The pooled estimate for the overall population was 0.068 (95% CI: 0.050–0.093), with high between‐study heterogeneity, as indicated by an I^2^ of 98.9% and *τ* ^2^ = 0.6481, suggesting that variability was not solely attributable to sampling error (Figure 2).

### 3.3 Meta‐Regression

Meta‐regression was performed to explore the association between study‐level characteristics and the proportion of DH cases. Among the variables tested, only the age group demonstrated a statistically significant effect. Specifically, pediatric studies reported significantly lower proportions compared to adult studies (*β* = −1.48, SE = 0.57, *p* = 0.01; 95% CI: −2.61; −0.36). Similarly, studies conducted in Northern Europe had a significantly lower estimate compared with the reference region (*β* = −0.93, SE = 0.39, *p* = 0.02, 95% CI: −1.70; −0.16). No significant associations were observed for publication year, mixed‐age populations, or other geographic regions (Table 3).

**TABLE 3:** Metaregression results based on the overall pooled analysis.

Variable	Level	Estimate	Std. Error	*p*‐value	95% CI
Year	Year	0.01	0.02	0.72	(−0.03, 0.04)
Age group	Mixed	−0.22	0.30	0.46	(−0.81, 0.37)
	Pediatrics	−1.48	0.57	0.01	(−2.61, −0.36)
Location	Northern Europe	−0.93	0.39	0.02	(−1.7, −0.16)
	Western Europe	0.18	0.52	0.73	(−0.84, 1.21)
	Eastern Europe	0.18	0.52	0.73	(−0.84, 1.2)
	Other	0.14	0.36	0.71	(−0.58, 0.85)

### 3.4 Subgroup Analysis

Stratified analyses were conducted to explore potential differences between subpopulations. In pediatric populations (*n* = 5 studies), the pooled proportion of DH among CD patients was 0.026 (95% CI: 0.014–0.045). In the 12 studies that contained only adult participants, the estimate was higher at 0.086 (95% CI: 0.058; 0.126). The difference between subgroups was statistically significant (Q‐test for subgroup differences, *p* = 0.0005), suggesting a higher occurrence of DH in adult celiac patients compared to pediatric cases (Supporting Information S1, Figure S1). Among sex‐specific studies, only five provided extractable male‐stratified data. Therefore, these sex‐specific estimates are based on a subset of included studies and are not directly comparable with the overall pooled prevalence. The proportion of DH in male‐only samples was estimated at 0.129 (95% CI: 0.070; 0.227), whereas in female‐only samples, the estimate rose to 0.096 (95% CI: 0.037; 0.229) (Supporting Information S1, Figure S2). The test for subgroup differences was not statistically significant (*p* = 0.6), indicating no evidence of a sex‐based difference in the prevalence of DH.

### 3.5 Sensitivity

In the sensitivity analysis excluding studies with unclear or non‐standardized outcome definitions, 17 studies were included, yielding a pooled prevalence of 0.063 (95% CI: 0.044–0.090), with high heterogeneity (I^2^ = 99.1%) (Supporting Information S1, Figure S3).

When studies conducted in selected populations were excluded, 18 studies were retained, and the pooled estimate was 0.067 (95% CI: 0.047–0.096), again with high heterogeneity (I^2^ = 99.1%) (Supporting Information S1, Figure S4).

Excluding studies based on registry or administrative data, 20 studies were retained, which resulted in a pooled prevalence of 0.088 (95% CI: 0.067–0.115), with slightly lower but still substantial heterogeneity (I^2^ = 89.5%) (Supporting Information S1, Figure S5).

Lebwohl et al. [16] contributed 49,744 out of 73,905 total participants (67% of the overall sample). However, under the random‐effects model, its statistical weight was 4.4% due to the high between‐study heterogeneity (I^2^ = 98.4%). In the sensitivity analysis excluding Lebwohl et al. [16], the pooled prevalence remained consistent at 0.078 (95% CI 0.057–0.103), based on 24,161 participants, confirming that the overall estimate was not driven by this single large study (Supporting Information S1, Figure S6). This also explains that the gap between the crude prevalence (3.0%) and the pooled random‐effects estimate (6.8%) reflects the dominant influence of the largest included study on the crude figure. Under high heterogeneity (*τ* ^2^ = 0.6481; I^2^ = 98.9%), the random‐effects model assigns more balanced weights across studies, giving relatively greater influence to smaller studies reporting higher DH proportions. This divergence underscores why random‐effects pooling is preferred over crude aggregation when heterogeneity is substantial.

### 3.6 Prevalence of DH in the General Population

The prevalence of DH in the general population has been explored in a limited number of population‐based studies conducted in different countries and healthcare settings. Data from four studies illustrate substantial geographic variation in incidence rates, which may reflect differences in diagnostic practices, genetic predisposition, and healthcare infrastructure. In Serbia, Milinkovic et al. [40] reported an incidence of 1.42 cases per million person‐year over 20 years using data from a dermatovenereology clinic register. In contrast, Smith et al. [41] found a higher incidence of 9.8 per million people per year (pmp/year) in Utah, USA, based on records from a hospital referral center and private dermatologists between 1978 and 1987. In the United Kingdom, West et al. [8] estimated the incidence at 12.3 pmp/year from 1990 to 2011 using data from the Clinical Practice Research Datalink, which captures primary care records. The highest incidence was reported in Finland by Salmi et al. [7], with 35.0 pmp/year from a specialized dermatology department registry spanning nearly 3 decades. Across studies, the female‐to‐male ratio ranged from 0.69 to 1.05, indicating a slight female predominance or near gender parity (Table 4).

**TABLE 4:** Incidence of dermatitis herpetiformis in the general population.

Authors and year of publication	Country	Time period (years)	Number of DH patients	Gender ratio F:M	Incidence (pmp/year)	Data source
Milinkovic, 2016 [40]	Serbia	1991–2010	156	0.77	1.42	Clinic of dermatovenereology register
Smith, 1992 [41]	Utah, USA	1978–1987	188	0.69	9.80	University of Utah hospital referral center register and private dermatologists
West, 2014 [8]	UK	1990–2011	809	1.05	12.30	Clinical practice research datalink (primary care in the United Kingdom)
Salmi, 2011 [7]	Finland	1980–2009	376	0.91	35.00	Department of dermatology, Tampere university hospital

Abbreviations: DH, dermatitis herpetiformis; F, female; M, male; pmp, per million population.

### 3.7 Risk of Bias (RoB)

Eight studies did not use appropriate diagnostic criteria for dermatitis herpetiformis. The remaining studies reported valid diagnostic approaches for DH, either based on biopsy confirmation or specialist clinical assessment (i.e., dermatologist‐documented DH diagnosis based on compatible clinical features, with direct immunofluorescence/biopsy when reported) (Supporting Information S1, Table S2).

In 7 studies (29%), RoB raised concerns regarding sample representativeness. For instance, Bottaro [20], Giorgetti [25], and Riznik [34] targeted subgroups such as subclinical CD patients, symptomatic children only, or excluded asymptomatic cases, potentially introducing selection bias. Similarly, 7 studies (29%) had unclear or no information on the DH diagnostic method, and it remained uncertain whether a standardized protocol was applied across all participants.

Concerning response rates, data were frequently missing or ambiguous: 10 studies (41,7%) either did not report response rates or failed to justify potential missing data or attrition.

Registry‐based studies posed an additional risk: 5 studies (21%) used administrative or registry data sources without clearly defining the reference population or time window for diagnosis (Grodé [13], Sorensen [36], Szaflarska‐Poplawska [37], Kotze [28], Lebwohl [16]). Among them, Grodé [13] and Kotze [28] were retained in sensitivity analyses only after confirming diagnostic clarity and completeness of stratification.

Visual inspection of the funnel plot suggested some asymmetry, particularly among smaller studies (Supporting Information S1, Figure S7). However, given the extreme between‐study heterogeneity (I^2^ ≈ 99%) and the use of logit‐transformed proportions, this pattern likely reflects true epidemiological variability and methodological differences rather than selective publication.

## 4 Discussion

This systematic review and meta‐analysis provides the most comprehensive estimate to date of the prevalence of DH among CD patients, as well as insights into the incidence of DH in the general population. Our findings suggest that approximately 6.8% (95% CI 5.0; 9.3%) of individuals with CD are diagnosed with DH, while the estimate was 6.7% (95% CI 4.7; 9.6) when analyses were restricted to non‐selected CD populations. Because most included cohorts were drawn from secondary care or referral settings, the pooled estimate may overrepresent symptomatic or complex CD cases and thus exceed the prevalence expected in unselected community cohorts.

The pooled prevalence aligns with historical evidence of a lower but clinically relevant burden of DH in CD cohorts, particularly in adults. Our estimate is consistent with previous reports suggesting a DH prevalence of 2%–8% among patients with biopsy‐confirmed CD [42, 43]. Subgroup and meta‐regression analyses showed that pediatric populations had a significantly lower prevalence (2.6%, 95% CI 1.4%–4.5%) compared to adults (8.6%, 95% CI 5.8; 12.6%), confirming prior observations that DH typically manifests in the third to fourth decades of life.

The heterogeneity observed across the studies was substantial (I^2^ = 98.9%, *τ* ^2^ = 0.6481) and persisted even after accounting for design and population characteristics. This suggests that heterogeneity reflects both true epidemiological variation (e.g., different CD phenotypes or referral patterns) and methodological differences, including inconsistencies in diagnostic protocol and data sources (e.g., clinical records, surveys, administrative data). Sensitivity analyses excluding studies with unclear diagnostic criteria, selected populations, or registry‐based data produced only minimal changes in pooled estimates (range: 0.06–0.09), indicating that these factors were not the primary drivers of variability. Moreover, restricting the analysis to studies reporting DH at the time of CD diagnosis did not materially reduce the pooled estimate. This suggests that the inclusion of studies reporting DH during follow‐up did not substantially inflate the overall prevalence and that the observed variability is more likely driven by differences in population characteristics, study design, and diagnostic approaches rather than timing of assessment.

Given the very high heterogeneity, the pooled prevalence should be interpreted as an average across diverse settings rather than a single generalizable estimate.

Although not the primary focus of this review, four additional studies [7, 8, 40, 41] assessed DH incidence in the general population, reporting values that ranged from as low as 1.42 [40] cases per million person‐years in Serbia to as high as 35.0 per million person‐years in Finland [7], with intermediate estimates of 9.8 in Utah, USA, and 12.3 in the UK. Such wide variation likely reflects a combination of genetic background, environmental exposures, and healthcare system factors, including differences in case‐finding intensity, access to dermatology services, and use of histological confirmation. Geographic differences in incidence parallel the known North–South gradient in CD prevalence and HLA‐DQ2/DQ8 distribution [44].

Publication bias and small‐study effects were explored through visual inspection of funnel plots. In meta‐analyses of proportions with extreme outcomes, funnel plot asymmetry may reflect statistical artifacts and true between‐study variability rather than true bias [18]. Therefore, any observed asymmetry was interpreted with caution. Formal statistical tests for funnel plot asymmetry were not performed due to the known methodological limitations of such tests in proportion meta‐analyses. When appraising small‐study effects, it is important to recognize that estimating the prevalence of DH was not the primary research aim in most of the included studies; DH frequency was usually reported as a secondary or incidental finding. This reduces the likelihood that studies were selectively published based on unusually high (or low) prevalence estimates.

## 5 Limitations

The included studies showed substantial heterogeneity in design, population characteristics, diagnostic criteria, and data sources, contributing to residual confounding despite the use of robust meta‐analytic methods such as random‐effects GLMM, subgroup, and meta‐regression analyses. Variability in DH diagnosis was a major concern: while CD was generally confirmed by biopsy or serology, several studies lacked clear or standardized criteria for DH, raising the risk of misclassification. Selection bias may also have occurred in studies focusing on subgroups, such as subclinical or symptomatic CD‐potentially leading to over, or underestimation of prevalence; this was addressed through sensitivity analyses.

In addition, some studies relied heavily on administrative or registry data [13, 16, 36] although valuable for large‐scale analyses, often lacked details on population definitions, diagnostic methods, or timing, limiting the reliability of reported rates. The wide temporal span of the included studies, from the 1960s to the 2010s, likely reflects evolving diagnostic standards and awareness, which could have influenced prevalence estimates. Finally, the limited representation of pediatric and non‐European populations restricts the generalizability of findings beyond adult European contexts.

## 6 Conclusions

This systematic review indicates that DH affects about 6.8% of CD patients, with a higher prevalence in adults. Although overall pooled prevalence appears to be declining, geographic differences persist. Findings underscore the need for standardized diagnostic criteria and more consistent reporting across clinical settings. Clinicians should promptly recognize DH lesions as they may represent a diagnostic clue for previously unrecognized CD and warrant appropriate gastroenterological evaluation. Further research should clarify DH epidemiology across different age groups and geographic settings, particularly in underrepresented populations.

## Author Contributions

H.O., F.Z., C.C.: conceptualization, H.O., I.B.: methodology, H.O., C.M.: data curation, G.B., F.Z.: investigation, H.O.: formal analysis, H.O.: visualization, C.C.: supervision, H.O., C.C.: resources, H.O., C.M.: writing – original draft, H.O., C.M., G.B., I.B., F.Z., C.C.: writing – review and editing, Guarantors of the article, H.O., C.C.. All authors approved the final version of the article.

## Funding

The authors have nothing to report.

## Ethics Statement

The authors have nothing to report.

## Conflicts of Interest

F.Z. has served as a speaker for Werfen, E.G. Stada Group, Fresenius Kabi, Kedrion, Janssen, Pfizer, Takeda, Unifarco, Malesci, Galapagos, AbbVie, Pharmaextracta, Lilly; F.Z. has served as a consultant for Galapagos, Takeda, and Tillotts Pharma. All other authors declare no conflicts of interest.

## Supporting information


Supporting Information S1

Supporting Information S2

## Data Availability

All data extracted and analyzed during this study are included in the main manuscript. These data are sufficient to allow for replication of the presented analyses.
