# Supplementary material for: Dermatitis Herpetiformis in Celiac Disease: A Systematic Review and Meta‐Analysis
Source: United European Gastroenterol J. 2026 Jul 7;14(6):e70259. doi: 10.1002/ueg2.70259 (PMC13338890; doi:10.1002/ueg2.70259)
Supplement: Supplementary file 1 — Supporting Information S1 [file UEG2-14-e70259-s002.docx]

**Supplementary File 1**

Manuscript title: *Dermatitis Herpetiformis in Celiac Disease: A Systematic Review and Meta-Analysis*

Authors: Honoria Ocagli¹, Chiara Monachesi¹, Giacomo Berti¹, Ileana Baldi¹, Fabiana Zingone²˒³, Cristina Canova¹

¹Unit of Biostatistics, Epidemiology and Public Health, Department of Cardio-Thoraco-Vascular Sciences and Public Health, University of Padova, Italy.
²Unit of Gastroenterology, Azienda Ospedale Università Padova, Padua, Italy.
³Department of Surgery, Oncology and Gastroenterology, University of Padua, Italy.

Summary

**[Tables](#_Toc231991692)** [2](#_Toc231991692)

[**Table S1** Search strategy 2](#_Toc231991693)

[**Table S2** Risk of bias 3](#_Toc231991694)

[**Figures** 4](#_Toc231991695)

[**Figure S1** Forest plot pediatric vs adults 4](#_Toc231991696)

[**Figure S2** Forest plot males vs females 5](#_Toc231991697)

[**Figure S3** Forest plot sensitivity excluding studies with unclear outcome definition 6](#_Toc231991698)

[**Figure S4** Forest plot sensitivity excluding studies conducted in selected populations 7](#_Toc231991699)

[**Figure S5** Forest plot sensitivity excluding registry-based (administrative) data 8](#_Toc231991700)

[**Figure S6** Forest plot sensitivity excluding the largest study (Lebwohl et al. 2020) 9](#_Toc231991701)

[**Figure S7** Funnel Plot overall 10](#_Toc231991702)

**[References](#_Toc231991703)** [11](#_Toc231991703)

# **Tables**

## **Table S1** Search strategy

| **Source** | **Search string** |
| --- | --- |
| PubMed | ("dermatitis herpetiformis"[MeSH Terms] OR "dermatitis herpetiformis"[Title/Abstract] OR "Duhring Disease"[Title/Abstract]) |
| Scopus | TITLE-ABS ("dermatitis herpetiformis" OR "Duhring Disease") |
| Embase | dermatitis herpetiformis.mp. or dermatitis herpetiformis/ or duhring disease.mp. |
| Web of science | dermatitis herpetiformis" OR "Duhring Disease" |

## **Table S2** Risk of bias

| **Author** | **Q1** | **Q2** | **Q3** | **Q4** | **Q5** | **Q6** | **Q7** | **Q8** | **Q9** |
| --- | --- | --- | --- | --- | --- | --- | --- | --- | --- |
| Bari (1) | Yes | Yes | Yes | Yes | Yes | Uc | Uc | Yes | Uc |
| Bottaro (2) | No | Yes | Yes | Yes | Yes | YES | No | Yes | Yes |
| Collin (3) | Yes | Yes | Yes | Yes | Yes | Yes | No | Yes | Uc |
| De Freitaas (4) | Yes | Uc | No | Yes | Yes | Uc | No | Yes | Uc |
| Delcò (5) | Yes | Yes | Yes | Yes | Yes | Yes | Yes | Yes | Yes |
| Dev (6) | Yes | Yes | Yes | Yes | Yes | Uc | Yes | Yes | Uc |
| Di Biase (7) | Yes | Yes | Yes | Yes | Yes | Yes | No | Yes | Yes |
| Giorgetti (8) | No | Yes | Yes | Yes | Uc | Yes | Yes | Yes | Yes |
| Grode (9) | Yes | Yes | Yes | Yes | Yes | Yes | No | Yes | Yes |
| Hauser (10) | No | Yes | Yes | Yes | No | No | No | Yes | No |
| Hawkes (11) | Yes | Yes | Yes | No | No | Yes | No | Yes | Uc |
| Khan (12) | Yes | Yes | No | Yes | Yes | No | Yes | Yes | Uc |
| Kotze (13) | Yes | Yes | Yes | Yes | Yes | No | Uc | Yes | Uc |
| Kotze (14) | Yes | Yes | Yes | Yes | Yes | No | Uc | Yes | Uc |
| Lebwohl (15) | Yes | Yes | Yes | Yes | Yes | Yes | No | Yes | Yes |
| Lima (16) | No | Yes | Yes | Yes | Yes | No | Uc | Yes | Uc |
| Papp (17) | Yes | Yes | Yes | Yes | Yes | Yes | No | Yes | Uc |
| Riestra (18) | No | Yes | No | Yes | Uc | No | Uc | Yes | Uc |
| Riznik (19) | No | Yes | Yes | No | No | Uc | Uc | Yes | Uc |
| Schiepatti (20) | No | Yes | Yes | Yes | Yes | Yes | Yes | Yes | Uc |
| Sorensen (21) | No | Yes | Yes | No | Yes | No | Yes | Yes | Yes |
| Szaflarska-Poplawska (22) | No | Uc | No | Yes | Uc | No | Yes | Yes | Uc |
| Volta (23) | Yes | Yes | Yes | No | Yes | Yes | Yes | Yes | Uc |
| Zingone (24) | Yes | Yes | Yes | Yes | Yes | Yes | Yes | Yes | Yes |

Q1. Was the sample frame appropriate to address the target population?
Q2. Were study participants sampled in an appropriate way?
Q3. Was the sample size adequate?
Q4. Were the study subjects and the setting described in detail?
Q5. Was the data analysis conducted with sufficient coverage of the identified sample?
Q6. Were valid methods used for the identification of the condition?
Q7. Was the condition measured in a standard, reliable way for all participants?
Q8. Was there appropriate statistical analysis?
Q9. Was the response rate adequate, and if not, was the low response rate managed appropriately?
Abbreviations: Uc = Unclear

# **Figures**

**Figure S1** Forest plot pediatric vs adults
Forest plot showing the pooled proportion of dermatitis herpetiformis among patients with celiac disease in pediatric and adult populations.


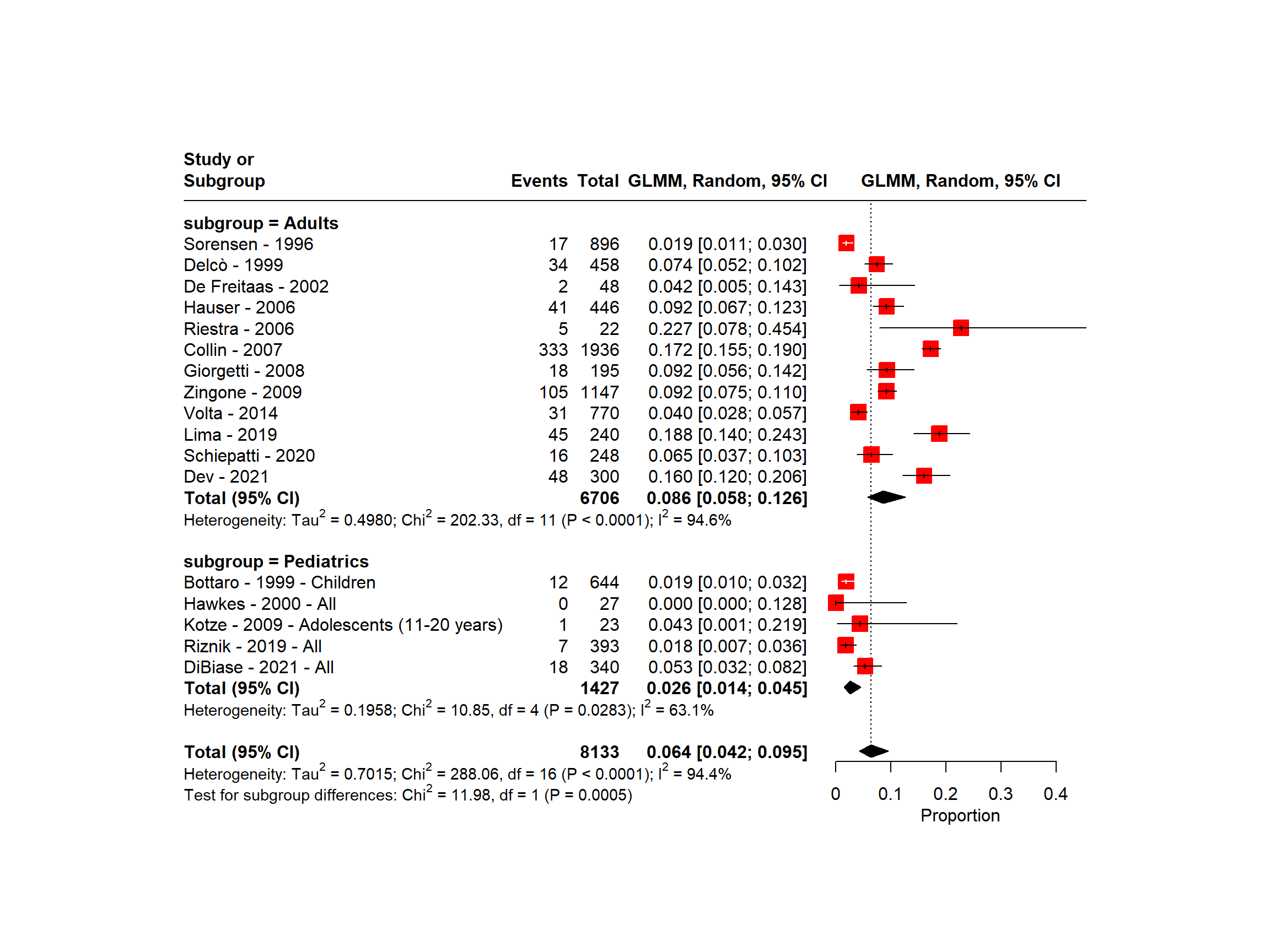


**Figure S2** Forest plot males vs females
Forest plot showing the pooled proportion of dermatitis herpetiformis among patients with celiac disease in males and females.


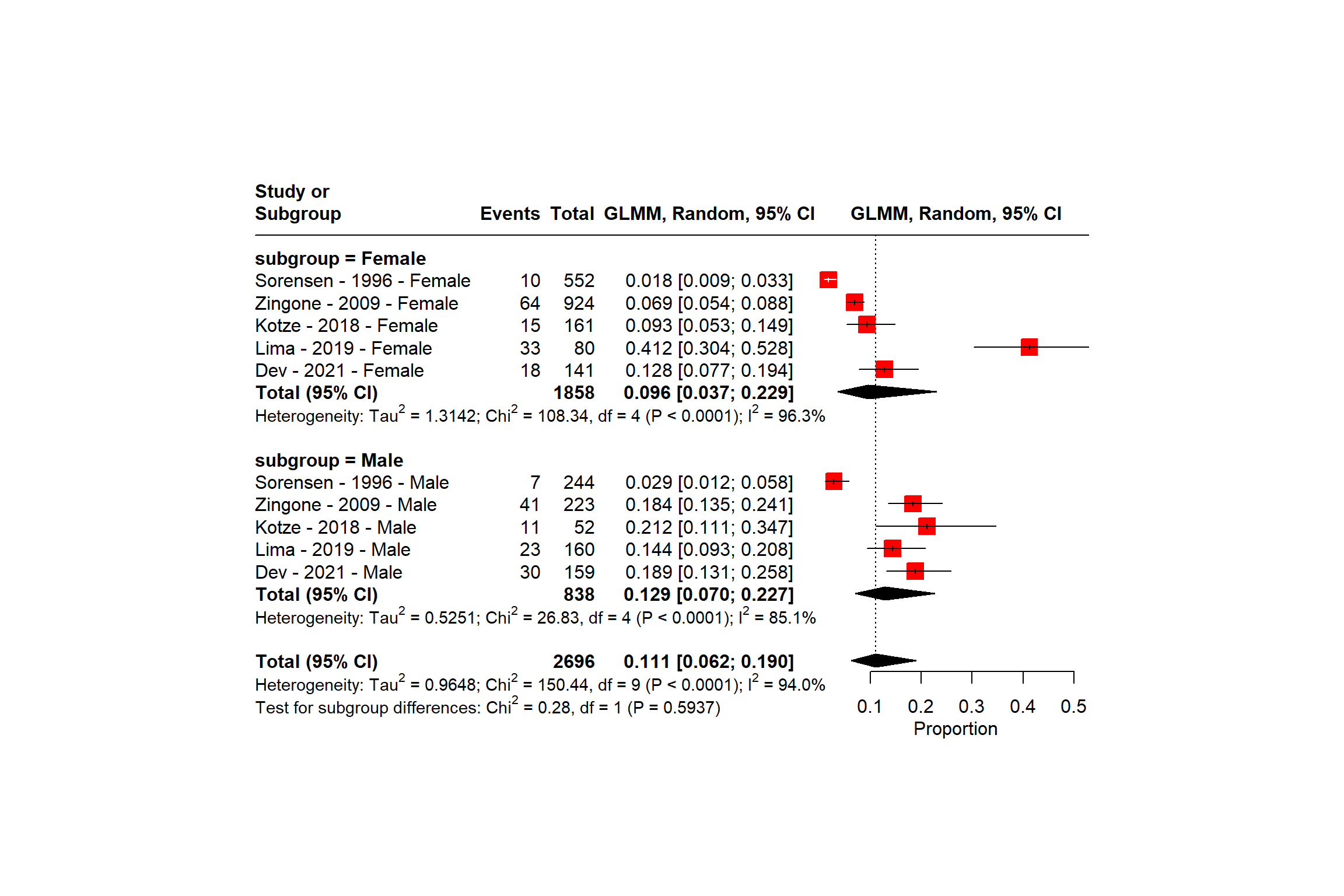


## **Figure S3** Forest plot sensitivity excluding studies with unclear outcome definition

Forest plot showing the pooled proportion of dermatitis herpetiformis among patients with celiac disease in the sensitivity analysis excluding studies with unclear or non-standardized outcome definition.


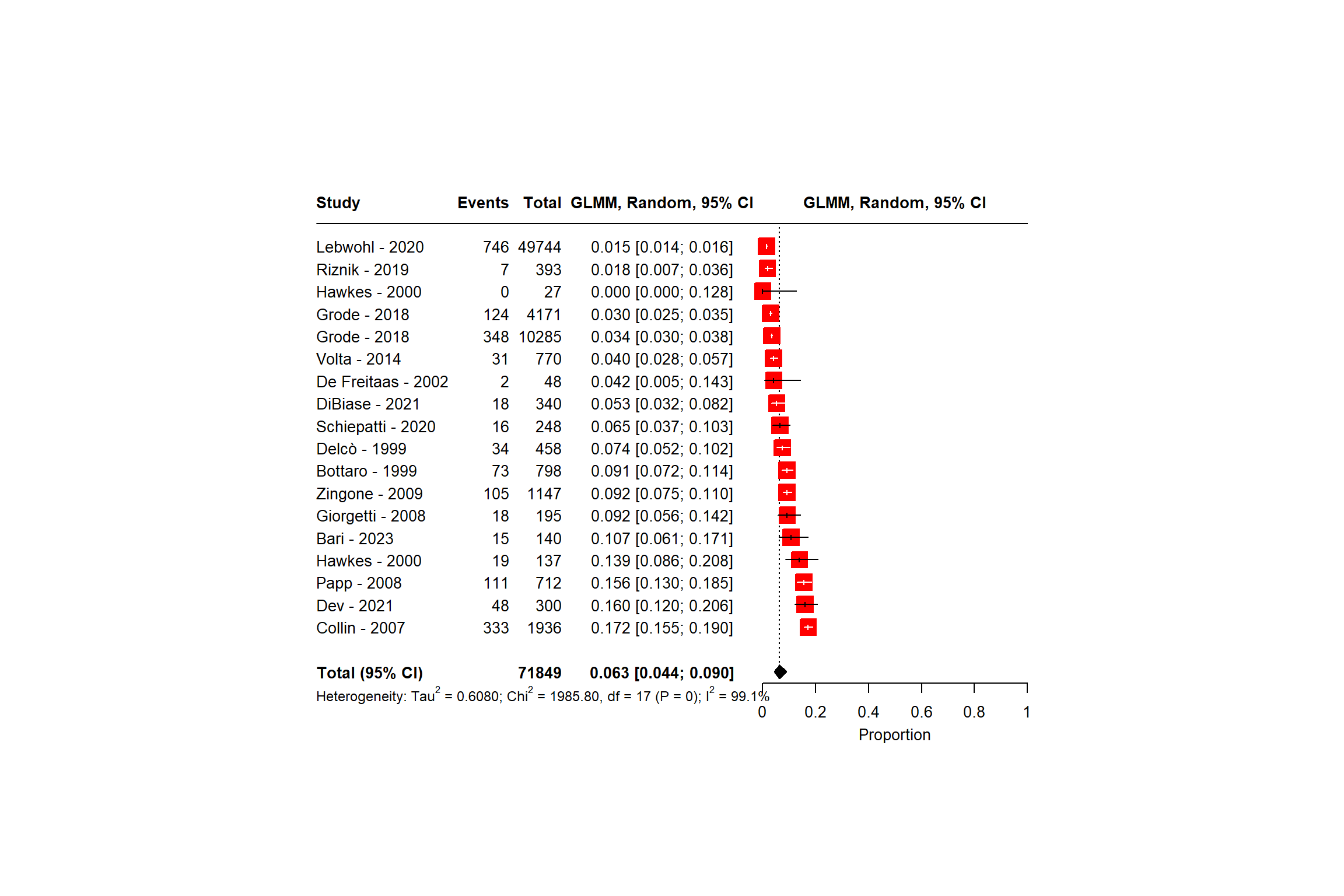


## **Figure S4** Forest plot sensitivity excluding studies conducted in selected populations

Forest plot showing the pooled proportion of dermatitis herpetiformis among patients with celiac disease in the sensitivity analysis excluding studies conducted in selected populations.


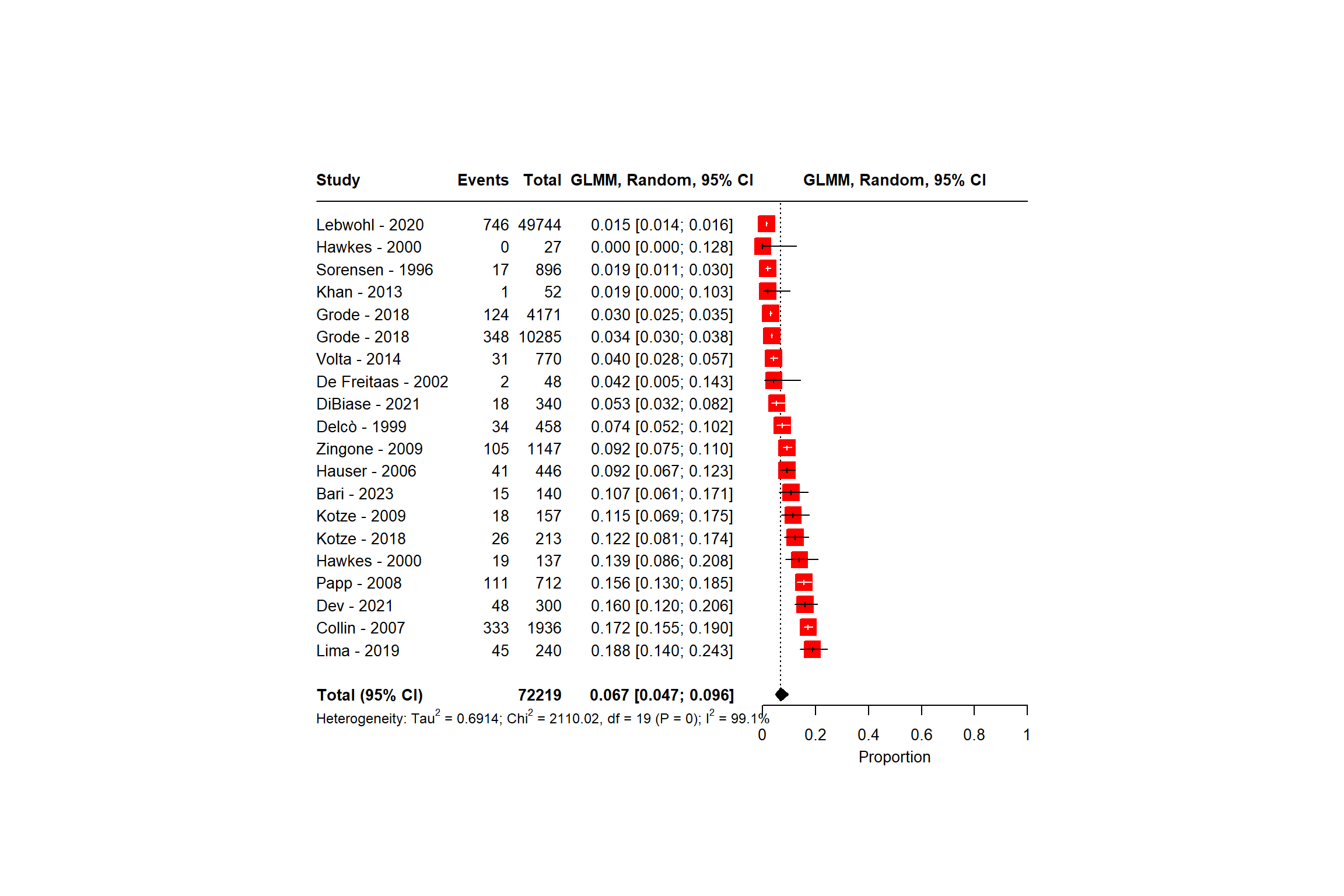


## **Figure S5** Forest plot sensitivity excluding registry-based (administrative) data

Forest plot showing the pooled proportion of dermatitis herpetiformis among patients with celiac disease in the sensitivity analysis excluding registry-based (administrative) data.


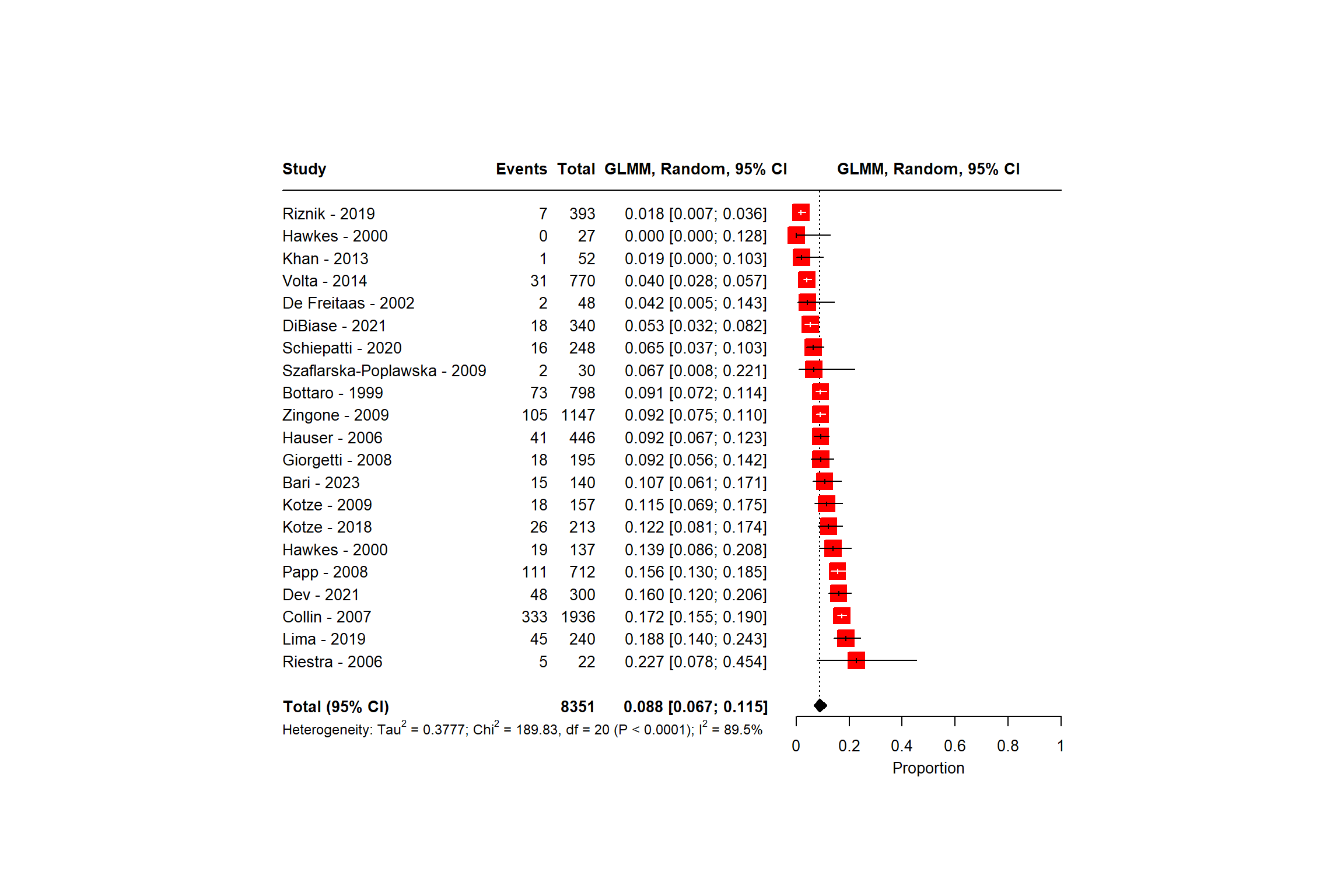


## **Figure S6** Forest plot sensitivity excluding the largest study (Lebwohl et al. 2020)

Forest plot showing the pooled proportion of dermatitis herpetiformis among patients with celiac disease in the sensitivity analysis excluding the largest study (Lebwohl et al.).


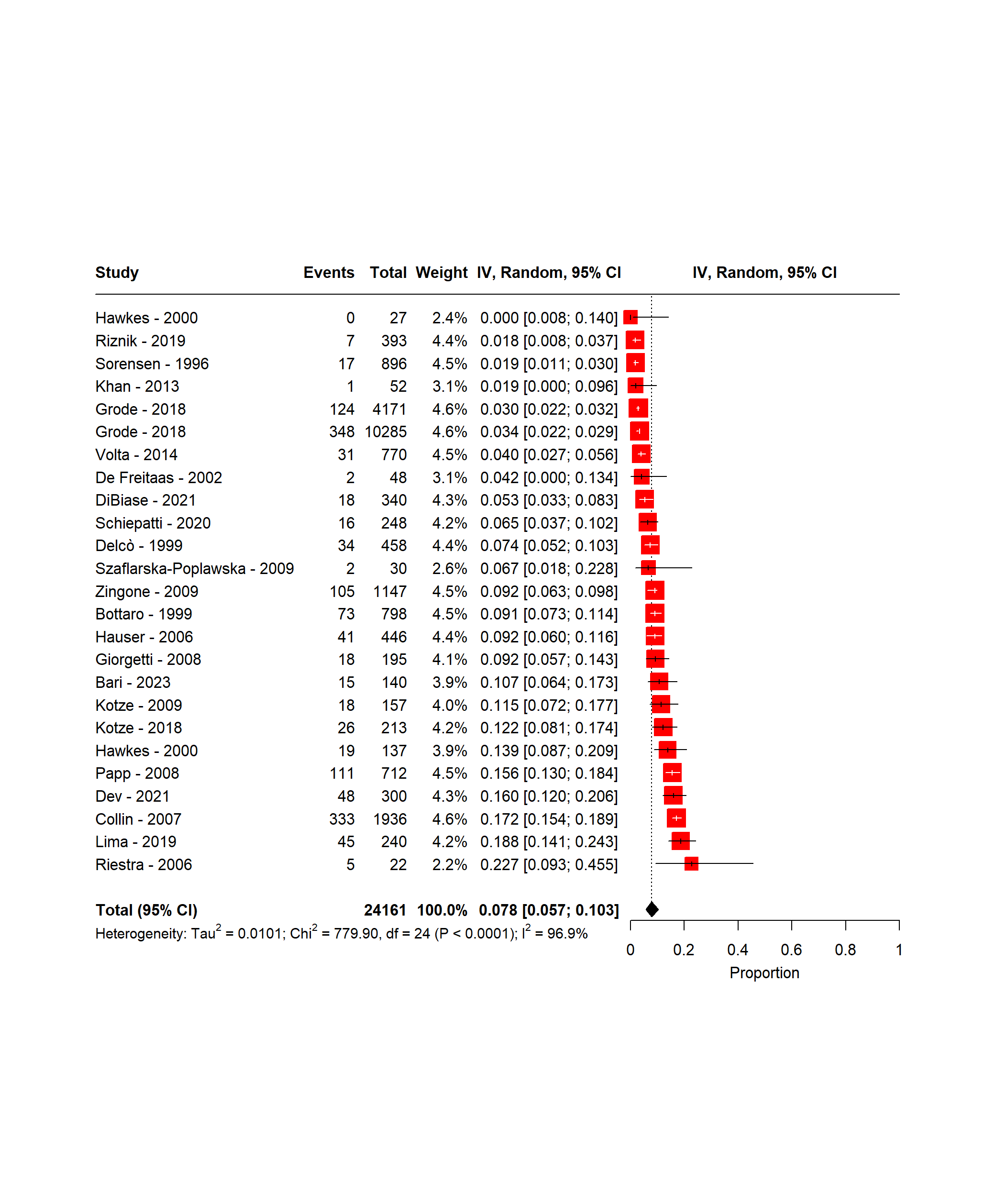


## **Figure S7** Funnel Plot overall


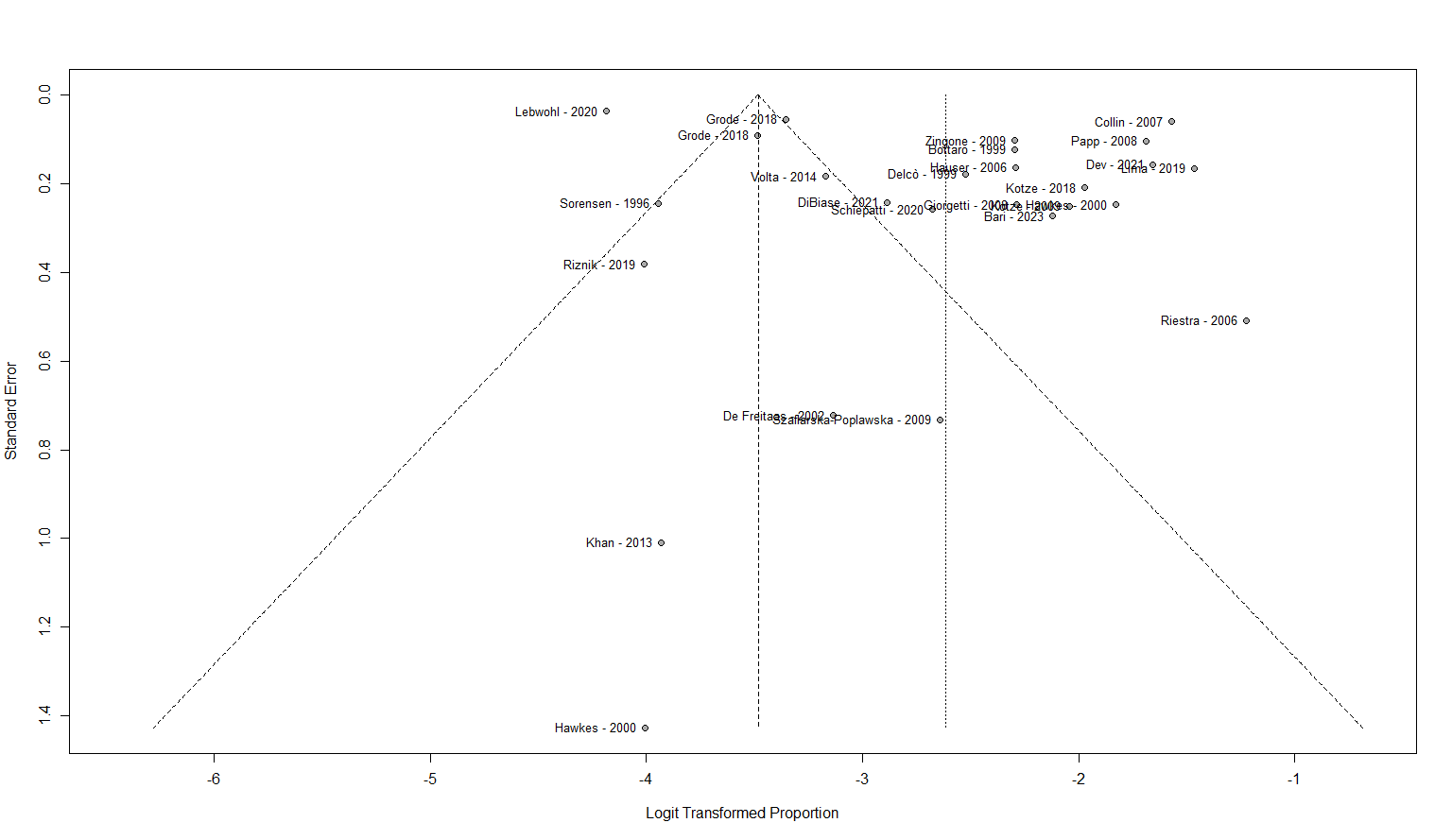


# **References**

1. Bari Z, Hadipour M, Fakheri H, Kazemi A, Maleki I, Taghvaei T, et al. Epidemiological, Endoscopic, Clinical, and Pathological Features of Patients with Celiac Diseases in Southern Littoral of Caspian Sea. Middle East J Dig Dis. 2023 Oct 30;15(4):257–62.

2. Bottaro G, Cataldo F, Rotolo N, Spina M, Corazza GR. The Clinical Pattern of Subclinical/Silent Celiac Disease: An Analysis on 1026 Consecutive Cases Figure 1. Am J Gastroenterol. 1999 Mar;94(3):691–6.

3. Collin P, Huhtala H, Virta L, Kekkonen L, Reunala T. Diagnosis of Celiac Disease in Clinical Practice: Physician’s Alertness to the Condition Essential. J Clin Gastroenterol. 2007 Feb;41(2):152–6.

4. De Freitas IN, Sipahi AM, Damião AOMC, De Brito T, Cançado ELR, Leser PG, et al. Celiac Disease in Brazilian Adults: J Clin Gastroenterol. 2002 Apr;34(4):430–4.

5. Delco F, El-Serag HB, Sonnenberg A. Celiac Sprue Among US Military Veterans (Associated Disorders and Clinical Manifestations). Dig Dis Sci. 1999 May;44(5):966–72.

6. Dev K, Mandhan R, Makheja K, Kumar J, Ahuja V, Ekta F, et al. Frequency of Cutaneous Disorders in Patients With Celiac Disease. Cureus [Internet]. 2021 Sept 21 [cited 2025 June 17]; Available from: https://www.cureus.com/articles/69314-frequency-of-cutaneous-disorders-in-patients-with-celiac-disease

7. Di Biase AR, Marasco G, Ravaioli F, Colecchia L, Dajti E, Lecis M, et al. Clinical Presentation of Celiac Disease and Diagnosis Accuracy in a Single-Center European Pediatric Cohort over 10 Years. Nutrients. 2021 Nov 18;13(11):4131.

8. Giorgetti GM, Lombardo M, Sandri G, Brandimarte G, Tursi A. A retrospective study of clinical manifestations of celiac disease: An 18 years review of activity in our Hospital. 2008. 18:54–60.

9. Grode L, Bech BH, Jensen TM, Humaidan P, Agerholm IE, Plana-Ripoll O, et al. Prevalence, incidence, and autoimmune comorbidities of celiac disease: a nation-wide, population-based study in Denmark from 1977 to 2016. Eur J Gastroenterol Hepatol. 2018 Jan;30(1):83–91.

10. H??user W, Gold J, Stein J, Caspary WF, Stallmach A. Health-related quality of life in adult coeliac disease in Germany: results of a national survey: Eur J Gastroenterol Hepatol. 2006 July;18(7):747–54.

11. Hawkes ND, Swift GL, Smith PM, Jenkins HR. Incidence and presentation of coeliac disease in South Glamorgan: Eur J Gastroenterol Hepatol. 2000 Mar;12(3):345–9.

12. DILARAM KHAN, ADNAN U REHMAN, SHER REHMAN. Celiac Disease: Clinical, Endoscopic and Histological Profile. Pak J Med Health Sci. 2013;7(NO.4 OCT – DEC 2013).

13. Kotze LMDS. Celiac disease in Brazilian patients: associations, complications and causes of death. Forty years of clinical experience. Arq Gastroenterol. 2009 Dec;46(4):261–9.

14. Kotze LMDS, Kotze LR, Moreno I, Nisihara R. IMMUNE MEDIATED DISEASES IN PATIENTS WITH CELIAC DISEASE AND THEIR RELATIVES: A COMPARATIVE STUDY OF AGE AND SEX. Arq Gastroenterol. 2018 Dec;55(4):346–51.

15. Lebwohl B, Söderling J, Roelstraete B, Lebwohl MG, Green PHR, Ludvigsson JF. Risk of skin disorders in patients with celiac disease: A population-based cohort study. J Am Acad Dermatol. 2021 Dec;85(6):1456–64.

16. Lima RF, Maria Da Silva Kotze L, Kotze LR, Chrisostomo KR, Nisihara R. Gender-Related Differences in Celiac Patients at Diagnosis. Arch Med Res. 2019 Oct;50(7):437–41.

17. Papp M, Foldi I, Nemes E, Udvardy M, Harsfalvi J, Altorjay I, et al. Haptoglobin Polymorphism: A Novel Genetic Risk Factor for Celiac Disease Development and Its Clinical Manifestations. Clin Chem. 2008 Apr 1;54(4):697–704.

18. Riestra S. Usefulness of duodenal biopsy during routine upper gastrointestinal endoscopy for diagnosis of celiac disease. World J Gastroenterol. 2006;12(31):5028.

19. Riznik P, De Leo L, Dolinsek J, Gyimesi J, Klemenak M, Koletzko B, et al. Diagnostic Delays in Children With Coeliac Disease in the Central European Region. J Pediatr Gastroenterol Nutr. 2019 Oct;69(4):443–8.

20. Schiepatti A, Maimaris S, Nicolardi ML, Alimenti E, Vernero M, Costetti M, et al. Determinants and Trends of Adherence to a Gluten-Free Diet in Adult Celiac Patients on a Long-term Follow-up (2000–2020). Clin Gastroenterol Hepatol. 2022 Apr;20(4):e741–9.

21. Sørensen HT, Fonager K. Risk estimation of disorders associated with coeliac disease. A 16-year Danish nationwide follow-up study based on hospital discharge data. Implications for screening. Int J Risk Saf Med. 1996;8(2):137–40.

22. Szaflarska-Popławska A. Patients with serological markers of coeliac disease but without features of atrophy concerning villi of the small bowel mucosa – own observations. 2009. 4((3)):152–8.

23. Volta U, Caio G, Stanghellini V, De Giorgio R. The changing clinical profile of celiac disease: a 15-year experience (1998-2012) in an Italian referral center. BMC Gastroenterol. 2014 Dec;14(1):194.

24. Zingone F, Bucci C, Tortora R, Santonicola A, Cappello C, Franzese MD, et al. Body Mass Index and Prevalence of Skin Diseases in Adults with Untreated Coeliac Disease. Digestion. 2009;80(1):18–24.
